# Editorial: Interspecific hybridization in plant biology

**DOI:** 10.3389/fpls.2022.1026492

**Published:** 2022-09-05

**Authors:** Dayun Tao, Ruslan Kalendar, Andrew H. Paterson

**Affiliations:** ^1^Yunnan Key Laboratory for Rice Genetic Improvement, Food Crops Research Institute, Yunnan Academy of Agricultural Sciences, Kunming, China; ^2^Helsinki Institute of Life Science HiLIFE, Biocenter 3, University of Helsinki, Helsinki, Finland; ^3^National Laboratory Astana, Nazarbayev University, Nur-Sultan, Kazakhstan; ^4^Plant Genome Mapping Laboratory, University of Georgia, Athens, GA, United States

**Keywords:** interspecific hybridization, crop improvement, synthetic polyploids, phenotypic variation, genomics, interspecific barriers, heterosis

Many crop gene pools are derived from small numbers of founders. As a consequence of long histories of strong directional selection, crop gene pools have narrow genetic diversity available to provide inherent solutions to changing needs or challenges. Notoriously, plants can mate across taxonomically-determined species boundaries, and interspecific hybridization is widely used in plant genetics research. Interspecific hybridizations have conferred practical improvements to crops, some of which are unexpected based on the phenotypes of the parents.

Genomics has provided insights into the fundamental consequences of interspecific hybridization for plant biology. Additionally, genomics has allowed the development of molecular tools for dissecting the genetic control of phenotypic variation in interspecific hybrid populations and manipulating interspecific introgressions in crop improvement.

This Research Topic aimed to publish peer-reviewed research in plant interspecific hybridization and its consequences, both fundamental and applied. While such work is prominent in plants, consideration will also be given to salient work in other taxa. A key threshold for publication was the extent to which findings are of cross-cutting interest and importance, i.e., not only to those working on the target taxon but also to a wide range of biological scientists. As a result, 15 articles were published.

For the issue of the role of interspecific hybridization, Wong et al. used the genus *Senecio*, and revealed some of the roles interspecific hybridization could play in evolution, including transcriptome shock, genome reorganization, change in mating system and reproductive traits, and adaptive introgression. Other aspects, such as the evolution of novel compounds, gene redundancy, and the extent of adaptive allele sharing, have been explored in other *Senecio* species. For cultivated species, Zhou et al. highlighted the important role that interspecific hybridization-introgression has played in improving the genetic diversity and adaptation of *Oryza sativa*. Natural hybridization-introgression is thought to have led to the origin of *indica, aus*, and *basmatic* subgroups, which adapted to changing cultivated environments, and produced feral weedy rice coexisting and competing with cultivars under production management. Artificial interspecific hybridization-introgression gained several breakthroughs in rice breeding, such as developing three-line hybrid rice, new rice for Africa (NERICA), and deploying some important pest and disease resistance genes in rice genetic improvement, contributing to the stable increase of rice production to meet the expanding human population.

For the issue of interspecific hybridization for breeding, Zhang et al. raised 6,372 agronomic trait introgression lines (ILs) from BC_2_ to BC_6_ based on the variations in agronomic traits by crossing 170 accessions of 7 AA genome species and 160 upland rice accessions of *O. sativa* as the donor parents, with three elite cultivars of *O. sativa*, Dianjingyou 1 (a *japonica* variety), Yundao 1 (a *japonica* variety), and RD23 (an *indica* variety) as the recurrent parents, respectively. Agronomic traits such as spreading panicle, erect panicle, dense panicle, lax panicle, awn, prostrate growth, plant height, pericarp color, kernel color, glabrous hull, grain size, 1,000-grain weight, drought resistance, and aerobic adaption, and blast resistance, were derived from more than one species. This agronomic trait introgression library from multiple species and accessions provided a powerful resource for future rice improvement and genetic dissection of agronomic traits. Tan et al. reconstructed the high-SER (stigma exsertion rate) trait based upon 18 quantitative trait loci (QTLs) for SER from *O. sativa, O. glaberrima*, and *O. glumaepatula* using single-segment substitution lines (SSSLs) in the genetic background of Huajingxian 74 (HJX74). A total of 29 pyramiding lines with 2-6 QTLs were developed from 10 SSSLs carrying QTLs for SER in the HJX74 genetic background. The results showed that the SER increased with increasing QTLs in the pyramiding lines. The SER in the lines with 5–6 QTLs was as high as wild rice with strong outcrossing ability. Limbalkar et al. used *Brassica carinata*-derived lines (CDLs) in *Brassica juncea* (L.) Czern. background, carrying genomic segments from *B. carinata*, to raise 105 hybrids developed from intermating 15 CDLs in half diallel fashion. The results indicated that higher productivity under drought conditions can be realized through the development of hybrids. Ullah et al. used *Trifolium occidentale*, one of the ancestral parents of *T. repens*, as a bridging species to overcome the interspecific barrier between *T. ambiguum* and *T. repens*. Recombinant chromosome segments from *T. ambiguum* were found in all five plants of *T. repens* background. Despite early chromosome imbalances, the backcross populations were fertile and produced large numbers of seeds. These hybrids represent a major new resource for the breeding of novel resilient forms of white clover.

Reproductive barriers, important in the wild to maintain species integrity, are a major obstacle to interspecific hybridization and gene introgression. Interactive RNA sequencing and proteome analysis by Du et al. revealed changes in the transcriptomic and proteomic profiles of *Fragaria viridis* styles harvested at 0 and 24 h after self-pollination. Differentially expressed genes and differentially abundant proteins associated with self-incompatible pollination were further mined, and multiple pathways were found to be involved. Moriyama et al. made a sibling cross of F_1_ plants made from the cross between *Lilium* × *formolongi* cv. Hatsuki and cv. Raizan 2go, producing the pollen-sterile line, PL01. Using PL01 and its progeny, genetic analysis suggested that the male-sterile phenotype is attributed to one recessive locus *LflflTDF1*. A transcript expressed only in pollen-fertile plants was homologous to *TDF1* (*DEFECTIVE in TAPETAL DEVELOPMENT and FUNCTION1*) in *Arabidopsis*, a gene encoding a transcription factor AtMYB35 known as a key regulator of pollen development. The *LflflTDF1* allele was transferred to Easter lily to confer sterility. He et al. reported that an imbalance in parental genomes or endosperm balance number (EBN) causes hybrid seed lethality and ovary abscission in both interspecific and intraspecific-interploidy crosses in the genus *Nicotiana*. Interesting, Kopecký et al. demonstrated that in *Allium cepa* × *A. roylei* hybrids, chromosomes of *A. cepa* are frequently substituted by those of *A. roylei* and in just one generation, the genomic constitution shifts from 8 *A. cepa* + 8 *A. roylei* chromosomes in the F_1_ generation to the average of 6.7 *A. cepa* + 9.3 *A. roylei* chromosomes in the F_2_ generation, inferring that female meiotic drive is the key factor underlying *A. roylei* genome dominance. Salina et al. identified regions of recombination suppression in wheat chromosome 5B based on comparisons of the 5B map of a cross between the Chinese Spring (CS) variety of hexaploid wheat and CS-5Bdic with several 5B maps of tetraploid and hexaploid wheat. Pan et al. reported that wheat- *Agropyron cristatum* derivative II-11-1 was proven to contain a pair of 5P chromosomes and a pair of 2P chromosomes with 42 wheat chromosomes by analyzing the fluorescence *in situ* hybridization (FISH) and expressed sequence tag (EST) markers. Additionally, cytological identification and field investigation showed that the 5P chromosome can weaken the homologous pairing of wheat chromosomes and promote pairing between homoeologous chromosomes. This provides new materials for studying the mechanism of the alien gene affecting homologous chromosome pairing and promoting homoeologous pairing of wheat.

The major issue is genomics of interspecific hybrids and hybrid heterosis. Mo et al. made a comprehensive comparative transcriptome sequencing analysis of root samples from the hybrid G70 × GDH11 and its parental inbred lines G70 and GDH11 to elucidate the importance of the root uptake capacity of potassium (K+) in the formation of heterosis in *Nicotiana tabacum* L. The results showed that 29.53% and 60.49% of the differentially expressed genes (DEGs) exhibited dominant and over-dominant expression patterns, respectively. Li et al. applied the reciprocal interspecific hybrids and their parents (*Gossypium hirsutum* and *Gossypium barbadense*) to elucidate the transcription regulatory mechanism of early biomass heterosis. Comparative transcriptome analysis showed that transgressive down-regulation (TDR) is the main gene expression pattern in the hybrids (*G. hirsutum* × *G. barbadense*, HB). Transgressive up-regulation (TUR) is the major primary gene expression pattern in the hybrids (*G. barbadense* × *G. hirsutum*, BH). The above results demonstrated that overdominance mediates biomass heterosis in interspecific hybrid cotton and the supervisory mechanism divergence of hybrids with different females. Cardoso-Silva et al. identified and characterized Orphan genes (OGs) in sugarcane. The results obtained suggested that sugarcane OGs are involved in several biological mechanisms, including stimulus response and defense mechanisms.

In summary, the research collected on this Research Topic facilitated understanding of issues related to interspecific hybridization in plant biology. We believe that this platform for enhancing exchange and promoting development has merit to be continued regarding some issues such as genomics of natural or synthetic polyploid formation, genomic responses to interspecific hybridization, transmission genetics across species boundaries, and genomics of interspecific hybrid heterosis.

## Author contributions

DT prepared the draft. All authors listed have made a substantial, direct, and intellectual contribution to the work and approved it for publication.

## Funding

This work was supported by National Natural Science Foundation of China (Grant No. 31991221) for DT and by the Ministry of Agriculture of the Republic of Kazakhstan in the framework of program funding for research (BR10765038) for RK.

## Conflict of interest

The authors declare that the research was conducted in the absence of any commercial or financial relationships that could be construed as a potential conflict of interest.

## Publisher's note

All claims expressed in this article are solely those of the authors and do not necessarily represent those of their affiliated organizations, or those of the publisher, the editors and the reviewers. Any product that may be evaluated in this article, or claim that may be made by its manufacturer, is not guaranteed or endorsed by the publisher.

